# Association between Platelet Transfusion and Delirium in Critically Ill Children

**DOI:** 10.3390/children10050825

**Published:** 2023-05-01

**Authors:** Matthew Nelligan, Marianne E. Nellis, Elizabeth A. Mauer, Linda M. Gerber, Chani Traube

**Affiliations:** 1 Morgan Stanley Children’s Hospital, NewYork-Presbyterian Columbia University, New York, NY 10032, USA; 2 Department of Pediatrics, Weill Cornell Medical College, New York, NY 10065, USA

**Keywords:** delirium, platelet transfusion, pediatric intensive care unit, inflammation, platelets

## Abstract

Delirium is a frequent, serious, and preventable complication in critically ill children. Inflammation has been implicated as a mechanism for the development of delirium. Platelet transfusions may potentiate the body’s pro-inflammatory responses. We hypothesized that receipt of platelets would be associated with delirium development in a pediatric intensive care unit (PICU). We performed a single-center retrospective cohort analysis including children admitted to the PICU between 2014 and 2018 who were transfused platelets within the first 14 days of admission. Data obtained included severity of illness, level of respiratory support, exposure to medications and blood products, as well as daily cognitive status. To account for time-dependent confounding, a marginal structural model (MSM) was constructed to delineate the relationship between platelet transfusion and next-day delirium. MSM demonstrated a 75% increase in the development of next-day delirium after transfusion of platelets (aOR 1.75, 95% CI 1.03–2.97). For every 1 cc/kg of platelet transfused, odds of next-day delirium increased by 9% (odds ratio 1.09, 95% CI 1.03–1.51). We reported an independent association between platelet transfusion and next-day delirium/coma after accounting for time-dependent confounders, with a dose–response effect. Minimizing platelet transfusions as much as clinically feasible may decrease delirium risk in critically ill children.

## 1. Introduction

Delirium is defined as an acute and fluctuating change in awareness and mentation that occurs in the setting of serious medical illness [1]. Delirium in adults with critical illnesses is well-described and associated with an increase in morbidity and mortality [2]. Pediatric research has shown that delirium is also a common complication of childhood critical illness affecting approximately 34% of all children admitted to the pediatric intensive care unit (PICU) [3]. Pediatric delirium has been linked to significant increases in hospital length-of-stay and posttraumatic stress symptoms [4,5,6]. Delirium is also independently associated with decreased quality of life and mortality in critically ill children [7,8,9]. By better understanding the modifiable risk factors associated with the development of delirium, providers may be able to decrease its incidence and minimize the associated morbidity.

Inflammation has been implicated as playing an etiologic role in delirium development in adult patients [10,11]. A systematic review of the studies on adults suggests that platelet transfusion potentiates the body’s pro-inflammatory processes [12]. Two recent studies called on a similar inflammation-based mechanism for the development of pediatric delirium and found that RBC transfusions were independently and temporally associated with delirium development [13,14]. Given the lack of current research surrounding platelet transfusion and delirium, it remains unknown whether platelet transfusions play a pathophysiologic role in the development of pediatric delirium.

Our study aimed to investigate the association between transfusion of platelets and development of delirium/coma in patients admitted to the pediatric intensive care unit. We also explored the presence of a dose–response relationship between the platelet volume transfused and development of delirium. We hypothesized that there was an independent association between transfusion of platelets and delirium development in children admitted to the PICU.

## 2. Materials and Methods

The Weill Cornell Medicine Institutional Review Board approved this study titled “Association Between Platelet Transfusions and Pediatric Delirium” (protocol 1807019459) on 16 January 2020. Procedures for the study were followed in accordance with the ethical standards of the Institutional Review Board on human experimentation and with the Helsinki Declaration of 1975. We utilized delirium data collected prospectively in an urban, academic, mixed pediatric critical care unit. Our study included all patients admitted to the PICU between 1 September 2014 through 31 December 2018 who were transfused platelets within 14 days of being admitted. It was well-known that patients who are transfused platelets are inherently “different” and more severely ill than patients who are not transfused platelets, and it is difficult to fully control for this difference. Therefore, rather than compare delirium rates in transfused and non-transfused patients, we elected to include only those patients who were transfused platelets. Granular daily data were collected in this cohort to allow for longitudinal analysis in order to investigate the temporal relationship between platelet transfusion and delirium development in these patients.

Patients were excluded if they did not receive platelet transfusions, were transfused in other units in the hospital, or if they did not have delirium screening performed during their PICU stay. The bedside nurse screened each patient, ages 0 to 21 years of age, once per shift (twice daily), utilizing the Cornell Assessment of Pediatric Delirium (CAPD) [15]. The CAPD score is a valid tool that is reliable for diagnosing delirium in critically ill children and infants, including those invasively mechanically ventilated. The operational definition of delirium for this study, consistent with published work involving delirium in pediatric patients, was a CAPD score greater than or equal to 9.

Patients were classified daily as either “comatose” (patients unable to arouse to verbal stimulation—commonly due to exposure to sedative medications and thus unable to detect delirium), “delirious” (patients with CAPD score ≥ 9), or “normal” (patients who do not have coma or delirium). The primary outcome measure of our study was presence of delirium and/or coma on the day following transfusion of platelets. We chose the outcome of delirium/coma as the primary endpoint because delirium could not be assessed in a comatose patient. In addition, delirium and coma are progressive levels of neurologic dysfunction. Our approach was derived from existing literature surrounding delirium, which demonstrated that patients who do not have delirium and/or coma (i.e.: delirium-free and coma-free, DFCF) had more favorable clinical outcomes.

Delirium was divided into three subtypes: hypoactive, hyperactive, and mixed delirium. The Richmond Agitation Sedation Scale (RASS) was utilized to assess the patient’s activity and agitation level [16]. If a delirious patient’s RASS score ranged from 0 to −3, their delirium was classified as hypoactive. Delirious patients were considered hyperactive if the RASS scores ranged from 0 to +4. Finally, delirious patients were labeled as having mixed delirium if their RASS scores crossed zero (including positive and negative numbers).

We collected information including admitting diagnoses, demographic data, and severity of illness at time of admission as calculated by Pediatric Index of Mortality-3 (PIM3) score [17]. Daily individual patient data obtained included RASS scores, CAPD assessments, respiratory support, and medications received. The data published involving platelet transfusion in this manuscript are novel without publication elsewhere.

Patients who received platelets during their PICU stay were identified via our institutional blood bank database. We obtained data surrounding transfusion including platelet dose (in cc/kg) as well as number of platelet transfusions (defined as number of days in the PICU in which a platelet transfusion was received). Patients who were transfused platelets were oftentimes transfused other blood products—including RBCs and plasma. As RBC transfusions are known to increase delirium risk, and little is known regarding the relationship between exposure to plasma and delirium, we additionally captured RBC and plasma exposure in all patients who were transfused platelets.

### Statistical Methods

Demographic and clinical data were reported as n (%) for categorical variables and as median (interquartile range [IQR]) or mean (standard deviation [SD]) for continuous variables. Univariate followed by stepwise multivariable logistic regression were used to assess the relationship between clinical interventions and development of delirium/coma and between platelet dose (exposure in cc/kg on days when platelets were transfused) and odds of next-day delirium/coma.

**Figure 1:**
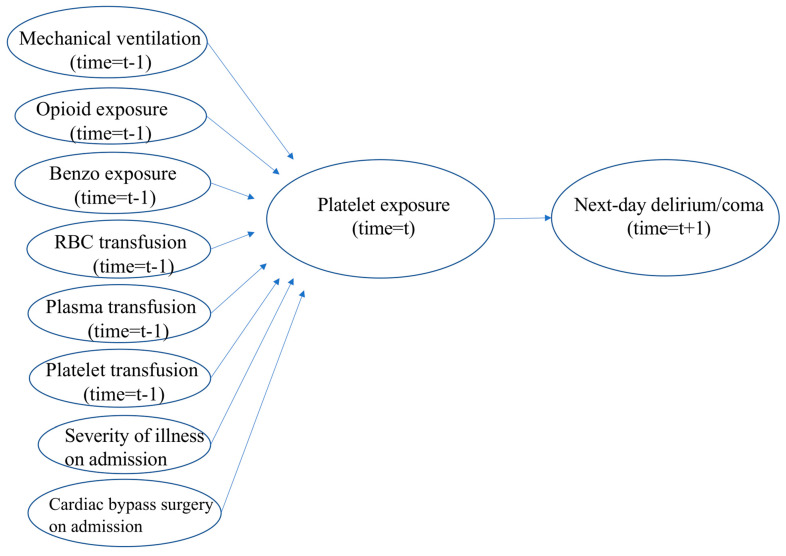
Schematic Representation of MSM Used to Account for Time Dependent Confounders in Relation to Receipt of Platelets and Subsequent Development of Delirium.

To account for time-dependent confounding, a marginal structural model (MSM) was constructed to assess the temporal effect of receiving a platelet transfusion on the odds of next-day delirium/coma [18]. As the receipt of platelets on a given day was influenced by other covariates, this model allowed for use of observational data to estimate causal effects of given exposures that varied with time. In this study, the odds of having a platelet transfusion were higher if the patient was severely ill, was on invasive mechanical ventilation, or was previously transfused RBCs. As all of these factors were themselves associated with delirium development, it was important to take these into account before attempting to evaluate for a potential independent and causal relationship between platelet transfusion and delirium development. To account for this confounding, two weight models were first produced. The first model was a logistic regression predicting receipt of platelets given severity of illness at PICU admission, history of cardiac bypass surgery, need for invasive mechanical ventilation, receipt of opioids, receipt of benzodiazepines, and prior blood product transfusions (Figure 1). As patient data were collected up to day 14 of admission, with some patients discharged prior to day 14, informative censoring was then modelled by a Cox proportional hazards regression. The same predictors were used as in model 1. Stabilized weights were produced from both models and multiplied to produce final weights. A final weighted logistic regression outcome model was constructed to evaluate the relationship between receipt of platelets and next-day delirium/coma. Sandwich standard errors were computed to take into account same-patient clustering.

The data were filtered to days with platelets administered and next-day delirium/coma status to assess the dose–response relationship between volume of platelets received and development of delirium/coma on the following day. A univariate logistic regression was constructed predicting odds of next day delirium/coma by cc/kg of platelets received.

There are two subgroups of PICU patients who are known to be at an extremely high risk for delirium: children under two years of age, and children who undergo cardiac bypass surgery. It is certainly possible that there are differential effects of possible risk factors (including platelet transfusions) in these particular groups. Therefore, although this single-center study was not powered for subgroup analyses, we also performed exploratory analyses on these high-risk patients.

All hypothesis testing was two-tailed, and a *p*-value < 0.05 was the threshold for significance. Analyses were performed in R Version 3.5.1 (R Foundation for Statistical Computing, Vienna, Austria).

## 3. Results

### 3.1. Clinical Characteristics of Patient Cohort and Platelet Transfusions

The cohort of patients analyzed included 213 PICU admissions (190 unique PICU patients) comprising 1744 PICU days. Table 1 describes the demographics of the included patients. A majority of patients were older than 5 years, and 60% were male. Twenty-eight patients were admitted after cardiac bypass surgery. Thirty-five percent of patients were mechanically ventilated on the day of admission. Overall, patients were on invasive mechanical ventilation for 45.6% (*n* = 796) of the 1744 PICU days, and the median length of PICU stay was 7 days. No patients were on extracorporeal membrane oxygenation. The mortality rate in the included patients was 11.7%.

Overall, patients were transfused platelets on 483 PICU days. All platelets were apheresed, 92% were irradiated, and 8% were pathogen reduced. The platelet dose per transfusion had a median of 9.6 cc/kg (IQR 5.2–14.0). Patients received a median of 1 platelet transfusion (IQR 1–2) during the study period. Nadir platelet levels in the cohort (lowest platelet level prior to platelet transfusion) had a median of 45 (IQR 20–91.5) × 10^9^/L. With respect to combined blood product exposure: on 159 days, patients also received RBCs; on 20 days, they also received plasma; and on 55 days, they received a combination of platelets, RBCs, and plasma.

### 3.2. Delirium Status

Overall, 41% of patients in this cohort were diagnosed with delirium. Daily delirium rate was 22% (*n* = 381 out of 1744 total PICU days). On 889 days (51%), patients were delirium free and coma free (DFCF). Daily data characteristics, which include the cognitive status and delirium subtype, are described in Table 2.

In univariate analyses, the following medical interventions were associated with the development of next-day delirium/coma: receipt of benzodiazepines or opioids, transfusion of any blood product, and transfusion of platelets or plasma or RBCs. In addition, invasive mechanical ventilation was strongly associated with higher-odds of developing next-day delirium (Table 3). It was noteworthy that odds of next-day delirium were further increased in children who were transfused plasma as well as platelets (OR 3.2, CI 1.9–5.4).

A marginal structural model allowed adjustment for time-dependent confounding. This model (as described in study methods) accounted for severity of illness and included exposure to cardiac bypass surgery, invasive mechanical ventilation, opioids, benzodiazepines, RBCs, plasma, and platelets. After accounting for the temporal relationship between all of these variables, there was a 71% increase in the odds of next-day delirium/coma after the transfusion of platelets (OR 1.71, 95% CI 1.004–2.900, *p* = 0.048) (Figure 1).

**Figure 2:**
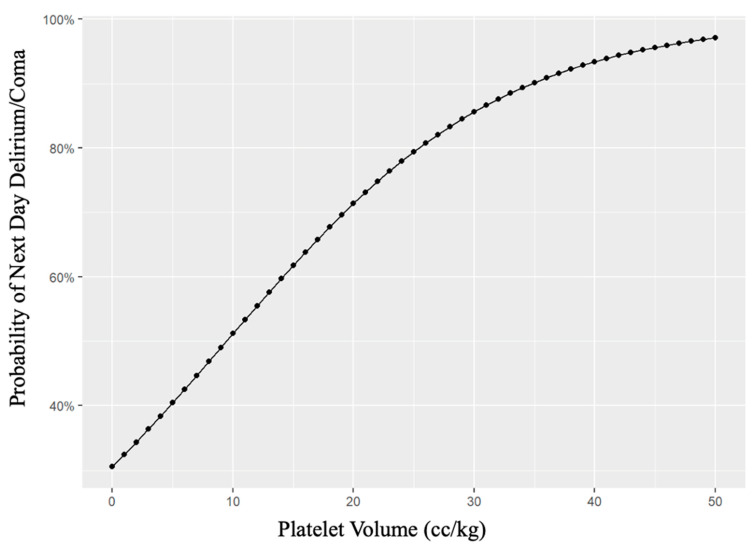
Dose–response Relationship Between Platelet Transfusions and Odds of Next-Day Delirium.

Finally, a regression model demonstrated that the odds of developing delirium or coma increased by 9% for each 1 cc/kg of platelets transfused (odds ratio 1.09, 95% CI 1.03–1.51, *p* = 0.002) (Figure 2).

In subgroup analyses, the results did not materially change. For children 0–2 years old, the marginal structural model demonstrated an odds ratio of 2.02 for next-day delirium/coma after the transfusion of platelets, with a trend towards significance (*p* = 0.057). The regression model showed that the odds of developing delirium or coma increased by 8% for each 1 cc/kg of platelets transfused (odds ratio 1.08, 95% CI 0.995–1.175, *p* = 0.06). For children who had undergone cardiac bypass surgery, the marginal structural model showed an odds ratio of 1.68 for next-day delirium/coma after the transfusion of platelets (95% CI 0.58–4.92, *p* = 0.34). With respect to the dose–response relationship, the regression model again demonstrated that the odds of developing delirium or coma increased by 9% for each 1 cc/kg of platelets transfused (odds ratio 1.09, 95% CI 0.968–1.229, *p* = 0.15).

## 4. Discussion

Our study was a novel single-center, observational examination of the relationship between receipt of platelet transfusion and development of delirium in a pediatric cohort. We demonstrated a significant positive association between platelet transfusion and development of next day delirium/coma—with a dose–response effect noted—after accounting for time-dependent confounders.

This finding is consistent with the limited existing literature. In a retrospective study of adult cardiothoracic surgery patients, intra-operative platelet transfusion was independently associated with development of delirium [19]. This is also consistent with the RBC transfusion literature, where several studies showed a strong and independent association between RBC transfusion and the development of pediatric delirium [13,14]. Authors suggested a two-hit mechanism for this relationship: critically ill children had an ongoing inflammatory response due to their underlying illness, and the blood product transfusion then multiplied this pre-existing inflammatory response.

Similarly, a review of the scientific literature suggested that platelet transfusions could induce a pro-inflammatory response in the recipient. Chen et al. suggested that elevated levels of platelet-derived microparticles (PMP’s) in stored platelet units may lead to a pro-inflammatory cascade in the transfused host. PMP’s are potent inflammatory mediators as they recruit immune cells such as NK-cells, T and B lymphocytes, and monocytes [12]. It is also interesting to note that patients in our cohort who received plasma in addition to platelets were at an even higher risk for delirium. Further studies are needed to delineate the role plasma particles play in inflammation and their subsequent contribution to delirium development.

From a patho-etiologic perspective, Ritter et al. described how inflammation could lead to alterations of the blood–brain barrier impairing cerebral blood flow and changing neurotransmitter balance. Their study also found that elevated serum inflammatory markers were associated with the occurrence of delirium in critically ill adults [10]. A prior study had also shown increased delirium rates in children with systemic inflammatory processes [7]. This was consistent with our overall hypothesis that inflammation played a large role in the etiology of pediatric delirium. Given the role of platelets as a pro-inflammatory mediator, it is intriguing to consider that the subsequent inflammatory cascade may have contributed to the pathologic process, by which children were at increased risk for developing delirium.

Our study findings are important because platelet transfusion represents a potentially modifiable risk factor for delirium development. Current pediatric literature demonstrates that platelet transfusions are most commonly given to nonbleeding patients as prophylaxis [20]. There is wide variability in platelet transfusion thresholds, which are often arbitrary and practitioner-dependent [20]. Studies in neonates have shown that using a higher prophylactic platelet transfusion threshold is not protective—in fact, it is associated with a higher incidence of major bleeding and excess mortality [21]. In addition, a recent study has shown an association between higher platelet transfusion thresholds in neonates and developmental disabilities at 2 years of age [22]. Intriguingly, the authors posit that this may be due to the pro-inflammatory effects of platelet transfusions, as a systemic inflammatory response has been associated with poor outcomes [23]. If, as our study suggests, platelet transfusions increase delirium risk, the delirium may mediate the subsequent neurodevelopmental issues. (This is consistent with the literature in adults, where delirium during critical illness is strongly associated with long-term cognitive decline in survivors) [24]. This would provide further support for utilizing restrictive strategies to limit platelet transfusions—and minimize delirium development—in the pediatric and neonatal ICUs [25].

Our study is limited by several factors. Most importantly, it is extremely important not to over-conclude based on the independent association between platelet transfusion and delirium described in this study. As an observational study, we cannot and do not establish causality. Environmental factors can contribute to delirium development (such as use of restraints, immobility, and ambient noise level), and we did not capture these factors in our dataset. Establishing an association between platelet transfusion and development of delirium is challenging, given sicker patients are transfused platelets more frequently. Although our cohort comprised exclusively patients who received platelets, and although we controlled for the severity of illness in our statistical investigations, this confounding is difficult to fully account for. Additionally, in this single-center study, we were not able to control all aspects of the platelet preparations (including storage age and ABO compatibility), nor were we powered to fully account for combined blood product exposures. As blood product transfusions are biologically active compounds—and pro-inflammatory contents of each transfusion likely differ based on donor, preparation, and storage methods—future large-scale studies should account for these characteristics and complex interactions. Finally, this study included patients in a PICU particularly focused on preventing delirium. The rates of delirium within the studied PICU are likely lower than rates of delirium in other PICUs. Given this fact, our findings may not be generalizable to all PICUs. Large multi-institutional studies are required to better understand the relationship between the receipt of platelet transfusions and the subsequent development of delirium.

## 5. Conclusions

Our study demonstrated an independent association between platelet transfusion and next-day delirium after controlling for time-dependent confounders, with a dose–response effect. As platelets are often transfused in the absence of clinical bleeding, and as platelet transfusion thresholds are often arbitrary, this may present a modifiable risk factor for delirium. Minimizing platelet transfusions as much as clinically feasible may decrease delirium risk in critically ill children.

## Figures and Tables

**Table 1:** Admission Characteristics.

Demographic and Clinical Characteristics	Admissions (n = 213)
Age Category (n, %)	
- 0–2 years - >2–5 years - >5–13 years - >13 years	59 (27.7%) 40 (18.8%) 57 (26.8%) 57 (26.8%)
Sex (male) (n, %)	127 (59.6%)
Invasive Mechanical Ventilation (n, %)	75 (35.2%)
Probability of Mortality (mean, SD)	6.2% (11.6%)
Admitting Diagnoses (n, %)	
- Cardiac Disease - Infectious/Inflammatory - Respiratory - Neurology - Hematologic/oncologic - Renal/metabolic	46 (21.6%) 46 (21.6%) 37 (17.4%) 36 (16.9%) 34 (16.0%) 14 (6.6%)
Hospital length of stay (days, median)	16
PICU length of stay (days, median)	7

**Table 2:** Daily Cognitive Status and Delirium Subtypes.

Daily Cognitive Status (*n*, %)	n = 1744 PICU days
Delirium/Coma-Free	889 (51.0%)
Delirium	381 (21.8%)
Coma	325 (18.6%)
Unknown	149 (8.5%)
**Delirium Subtype (*n*, %)**	**n = 381 days with delirium**
Hypoactive	279 (73.2%)
Mixed	88 (23.1%)
Hyperactive	14 (3.7%)

**Table 3:** Medical Interventions and Univariate Association with Next-Day Delirium.

Intervention	Odds of Next-Day Delirium/Coma (OR, 95% CI)
Invasive Mechanical Ventilation	OR 27.34, 95% CI (15.56–48.20)
Receipt of benzodiazepines	OR 3.46, 95% CI (2.32–5.16)
Receipt of opioids	OR 4.26, 95% CI (2.53, 7.16)
Any blood product transfusion	OR 2.04, 95% CI (1.47–2.82)
RBC transfusion	OR 1.62, 95% CI (1.20–2.19)
Platelet transfusion	OR 1.81, 95% (1.28–2.56)
Plasma transfusion	OR 3.20, 95% CI (1.89–5.41)

## Data Availability

Data are included within the article.
